# Genetic Analysis of Cytokine Promoters in Nonhuman Primates: Implications for Th1/Th2 Profile Characteristics and SIV Disease Pathogenesis

**DOI:** 10.1080/10446670410001670472

**Published:** 2004-03

**Authors:** Pavel Bostik, Melanie Watkins, Francois Villinger, Aftab A. Ansari

**Affiliations:** 1 Department of Pathology and Laboratory Medicine Emory University 1639 Pierce Drive Atlanta GA 30322 USA

## Abstract

The shift from a predominant synthesis of prototype Th1 cytokines to Th2 or Th0 type of cytokines by antigen activated PBMC's from HIV infected humans and SIV infected disease susceptible rhesus macaques (RM) has been shown to be associated with disease progression. Paradoxically, antigen activated PBMC's from sooty mangabeys (SM), which are naturally infected with SIV and are disease resistant despite high viral loads, maintain a predominant Th2 cytokine profile. It has been reasoned that the resistance to perturbations of cytokine synthesis by slow and/or nonprogressor HIV infected patients and SIV infected disease susceptible RM is secondary to inherited polymorphisms within the promoter regions for cytokines. Similar promoter polymorphisms could also contribute to the cytokine profile of PBMC's from SM. To address this issue promoter regions for the major Th1/Th2 cytokines from RM and SM were cloned and sequenced. Sequence analysis of promoter fragments of IL-4, IL-10, IL-12 p40, IFN-gamma and TNF-alpha from the two monkey species showed varying degree of homology ranging from high degree of homology detected for IFN-gamma promoter (>99%) to relatively high degree of polymorphism detected for TNF-alpha promoter (94% homology). In addition, several variable regions within the promoters of IL-12 p40, IL-10 and TNF-alpha in the two species contain polymorphisms in sequences that constitute binding sites of known transcription factors (TF). Such differences are likely to differentially bind TF and thus either qualitatively and/or quantitatively affect the regulation of cytokine synthesis in these two species and potentially contribute to disease progression and/or resistance.


*Clinical & Developmental Immunology*, March 2004, Vol. 11 (1), pp. 35–44

Taylor & Francis healthsciences

### Genetic Analysis of Cytokine Promoters in Nonhuman Primates: Implications for Th1/Th2 Profile Characteristics and SIV Disease Pathogenesis

PAVEL BOSTIK*, MELANIE WATKINS, FRANCOIS VILLINGER and AFTAB A. ANSARI

*Department of Pathology and Laboratory Medicine, Emory University, 1639 Pierce Drive, Atlanta, GA 30322, USA*

The shift from a predominant synthesis of prototype Th1 cytokines to Th2 or Th0 type of cytokines by antigen activated PBMC's from HIV infected humans and SIV infected disease susceptible rhesus macaques (RM) has been shown to be associated with disease progression. Paradoxically, antigen activated PBMC's from sooty mangabeys (SM), which are naturally infected with SIV and are disease resistant despite high viral loads, maintain a predominant Th2 cytokine profile. It has been reasoned that the resistance to perturbations of cytokine synthesis by slow and/or nonprogressor HIV infected patients and SIV infected disease susceptible RM is secondary to inherited polymorphisms within the promoter regions for cytokines. Similar promoter polymorphisms could also contribute to the cytokine profile of PBMC's from SM. To address this issue promoter regions for the major Th1/Th2 cytokines from RM and SM were cloned and sequenced. Sequence analysis of promoter fragments of IL-4, IL-10, IL-12 p40, IFN-gamma and TNF-alpha from the two monkey species showed varying degree of homology ranging from high degree of homology detected for IFN-gamma promoter (>99%) to relatively high degree of polymorphism detected for TNF-alpha promoter (94% homology). In addition, several variable regions within the promoters of IL-12 p40, IL-10 and TNF-alpha in the two species contain polymorphisms in sequences that constitute binding sites of known transcription factors (TF). Such differences are likely to differentially bind TF and thus either qualitatively and/or quantitatively affect the regulation of cytokine synthesis in these two species and potentially contribute to disease progression and/or resistance.

*Keywords*: Promoter; Cytokine; Nonhuman primate; SIV

### INTRODUCTION

Both CD4+and CD8+ T cells mediate their biological effect upon recognition of their cognate peptide-MHC ligands in part by the synthesis of a spectrum of cytokines. The quality and quantity of immune response generated is in part governed by the spectrum of these cytokines. It has been previously reported that disease progression in HIV infected humans leads to a "shift" in the cytokine expression profile from a Th1 to Th2 prototype characterized by defective production of interferon gamma (IFN-gamma), IL-2 and IL-12 with a concomitant increase in production of IL-4, IL-5, IL-6, and IL-10 (Klein *et al.*, 1997) while the maintenance of the Th1 type cytokine secretion pattern was found characteristic of slow disease progression in the long term nonprogressor (LTNP) HIV infected humans (Clerici *et al.*, 1996). Other study has however shown that PBMC from HIV infected patients show a decreased ability to produce both Th1 (IFN-gamma) and Th2 (IL-4) cytokines and that high frequency of clones generated from these cells exhibited a Th0 phenotype (producing both Th1 and Th2 type cytokines) that enhanced HIV replication (Maggi *et al.*, 1994). Similarly in SIV disease susceptible nonhuman primates—such as cynomolgous macaques—pathogenic SIV infection induces predominantly Th2 prototype cytokines (Benveniste *et al.*, 1996a) and an increase in Th1 prototype cytokine IFN-gamma levels was associated with better control of SIV infection with attenuated, but not fully pathogenic virus (Benveniste *et al.*, 1996b). In vaccination experiments induction of IFN-gamma production has been shown to correlate with protection to rechallenge with pathogenic virus (Ahmed *et al.*, 2002).

Natural SIV infection of nonhuman primates has become an important model and a useful tool for studies of the pathogenesis of lentivirus induced disease. The fact that naturally SIV infected species—such as sooty mangabeys (Cercocebus atys) (Fultz *et al.*, 1986)—do not develop SIV induced disease despite viral loads and virus replication levels comparable to those in SIV

*Corresponding author. Tel.: +1-404-712-2835. Fax: +1-404-712-1771. E-mail: pbostik@emory.edu

ISSN 1740-2522 print/ISSN 1740-2530 online © 2004 Taylor & Francis Ltd
DOI: 10.1080/10446670410001670472

infected disease susceptible species makes this model especially attractive for studies aimed at defining the immune response mechanisms important for the "containment" as compared with development of the disease. Interestingly, during the studies of Th phenotype of CD4+ T cells from sooty mangabeys (SM), it was discovered that despite the lifelong lack of SIV disease T cell clones derived from both the SIV seronegative and seropositive animals continuously demonstrate predominantly a Th2 (or Th0) phenotype (Ansari *et al.*, 1994). This predominantly Th2/Th0 response observed in SM irregardless of SIV status therefore represents an interesting phenomenon since it is associated with disease resistance rather than with accelerated disease progression.

It has been hypothesized that one of the mechanisms underlying the potential resistance to the lentivirus induced perturbations in the expression of cytokines synthesized by T cells could be due to genetically inherited polymorphisms within the promoter regions for these cytokines. However, studies aimed at defining promoter sequences in HIV infected patients with diverse clinical stages produced controversial results. While some studies have failed to demonstrate a link between select polymorphisms in cytokine promoters for TNF-alpha (Brinkman *et al.*, 1997; Knuchel *et al.*, 1998) and IFN-gamma (Bream *et al.*, 2000) and disease progression in HIV infected patients, others have shown an association of the −308A polymorphism in the TNF-alpha promoter with AIDS dementia (Quasney *et al.*, 2001) and −589T polymorphism in the IL-4 promoter with an accelerated switch from a NSI to a SI phenotype of HIV-1 and potentially faster disease progression (Nakayama *et al.*, 2000).

Our studies with the nonhuman primate model of AIDS prompted us to identify potential polymorphisms in cytokine promoter sequences that may play a role in the lentivirus infection and disease. Here we analyze promoter sequences of the cytokines characteristic for Th1 and Th2 prototype response from both SIV disease susceptible (rhesus macaque) and SIV disease resistant (sooty mangabey) nonhuman primate species with the aim to identify those that may underlie the differences in both Th phenotype and SIV disease susceptibility observed in these two species.

### MATERIALS AND METHODS

### Cells

The peripheral blood samples were obtained from normal healthy adult rhesus macaques (Macaca mulatta) and adult healthy SIV seronegative and seropositive sooty mangabeys (Cercocebus atys) housed at the Yerkes Regional Primate Research Center of Emory University. All animals were maintained according to the guidelines of the Committee on the Care and Use of Laboratory Animals of the Institute of Laboratory Animal Resources, National Research Council and the Health and Human Services guidelines "Guide for the Care and Use of Laboratory Animals." Peripheral blood mononuclear cells (PBMC) were isolated using Lymphocyte Separation Medium (Cellgro, Herndon, VA).

### Cloning of Promoter Sequences

DNA was isolated from PBMC samples using the Wizard Genomic DNA purification kit (Promega, Madison, WI). Promoter sequences were amplified utilizing primer pairs as follows: 5′-GTGGCTCACACCTGTAATC-3′ and 5′-AGTTTATCAGGAGAAGCTAACGAT-3′ for IL-4 promoter; 5′-TTTGGGAAGGGGAAGTAGGG-ATAG-3′ and 5′-GGAGGACCAGGCAACAGAGC-AGTG-3′ for IL-10 promoter; 5′-GCGTCTAGAGTGCC-ATCCAAAGTGTTGTA-3′ and 5′-GAGGAATTCACAA-TGTGCTGCACCTCCTCT-3′ for IFN-gamma promoter; 5′-TATGAATTCCTGTATGCCTCCCTGAGGG-3′ and 5′-TGTTCTAGACTGCTGTTGCTGGTACTGG-3′ for IL-12 p40 promoter; 5′-TGAGGAATGGGTTACAGGA-3′ and 5′-CCTCTGCTGTCCTTGCTGA-3′ for TNF-alpha promoter. Amplification was performed using Advantage2 PCR kit (Clontech, Palo Alto, CA) in 25 cycles (95 for 30 s, 58° for 90 s). PCR products were digested with XbaI and EcoRI restriction enzymes and cloned into pGEM-7 (for IL-12 p40 and IFN-gamma promoter sequences) or directly cloned into pGEM-T vector (both Promega) and sequenced using SP6 and T7 primers. Sequences were analyzed using DNASTAR analysis package and putative transcription factor (TF) binding sites were located using TFSEARCH software (Y. Akiyama, RWCP, Japan) and TRANSFAC database (Heinemeyer *et al.*, 1998). Sequences from samples from two donors of each species were analyzed independently and consensus sequence was generated. Nucleotide sequence data reported are available in the GenBank database under the accession numbers: AY486428-AY486436.

### IL-4 and IFN-gamma Bioassay

PBMC were cultured in RPMI 1640 (Life Sciences, Gaithesburg, MD) media supplemented with 10% fetal calf serum (Cellgro), L-glutamine and gentamycin (both Life Sciences). Cells were stimulated with 10 μg/ml of phytohemagglutinin (PHA-P, DIFCO, Detroit, MI) for 48 h and supernatants analyzed for the cytokines as previously described (Villinger *et al.*, 1993).

### RESULTS AND DISCUSSION

### T Helper Profile in Nonhuman Primates

Results of previous studies have suggested that the spectrum of T helper cell response based on the synthesis of cytokines plays an important role in the outcome of

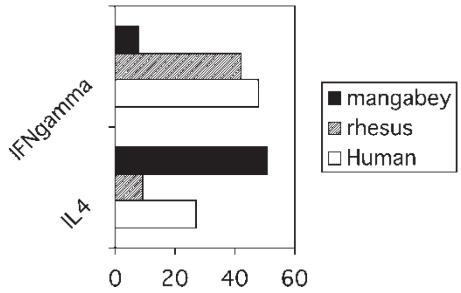

FIGURE 1 Expression of Th1/Th2 cytokines by PBMC from nonhuman primates and humans. PBMC from human ($n = 6$), RM ($n = 10$) and SM ($n = 10$) were stimulated with PHA-P and supernatant fluids assayed for the levels of IL-4 and IFN-gamma by bioassay as previously described (Villinger *et al.*, 1993).

infectious diseases including AIDS. An increase in the prototype Th2 cytokines—such as IL-10 and IL-4—and decrease in the prototype Th1 cytokines—such as IFN-gamma and IL12—was shown to be associated with infection and/or with rapid disease progression in HIV infected humans (Stylianou *et al.*, 1999) and SIV infected macaques (Benveniste *et al.*, 1996a). Our previous analysis of random cloned cell lines from rhesus macaques (RM) and SM showed that, interestingly, the majority of the T cell clones (including CD4+, CD8+ and double positive) from SM exhibit a Th2 phenotype (62 of 93 total) while a majority of similar clones derived from RM exhibit a Th1/Th0 phenotype (80 of 89 total) regardless of SIV status (Ansari *et al.*, 1994). Analysis of the IL-4 and IFN-gamma levels (major Th2 and Th1 cytokines, respectively) produced by *in vitro* PHA stimulated PBMC cultures from RM and SM show (Fig. 1) that, indeed, the SM cells produce significantly higher level of IL-4 (Th2) while the RM cells (similar to human cells) produce significantly higher levels of IFN-gamma.

The finding that RM which are SIV induced disease susceptible synthesize quantitatively higher levels of IFN-gamma, a prototype Th1 cytokine, and SM who are SIV infection disease resistant synthesize relatively higher levels of IL-4, a prototype Th2 cytokine, presents a paradox. It is reasonable to assume that in either case the unique spectrum of cytokines synthesized by PBMC's from each of the two species must be—at least in part—regulated at the transcriptional level by TF and therefore influenced by potential polymorphisms within the promoter regions of the cytokines that may be unique for the two species. The promoter regions for the cytokines were therefore amplified, sequenced and a comparative analysis performed.

### IL-12 p40 Promoter

IL-12 is a major cytokine driving the differentiation of naïve T cells into the Th1 phenotype (Manetti *et al.*, 1993; Mingari *et al.*, 1996). It is a heterodimer consisting of two subunits—p40 and p35 (Gubler *et al.*, 1991). A significant decrease in the production of IL-12 was observed in PBMC samples from HIV-1 infected patients that was not

TABLE I Cytokine promoter sequence pair distances

| | Length (bp)* | RM vs. HU[†] (%) | SM vs. HU (%) | RM vs. SM (%) |
|---|---|---|---|---|
| IFN-gamma | 750 | 97.3 | 97.2 | 99.6 |
| IL-12 p40 | 731 | 95.7 | 95.7 | 99.0 |
| IL-10 | 1171 | 96.8 | 96.5 | 99.1 |
| IL-4 | 996 | 96.4 | 96.9 | 99.0 |
| TNF-alpha | 999 | 94.4 | 93.9 | 98.8 |

* Length of analyzed sequence.
[†] Percent similarity between sequences from rhesus macaque (RM), human (HU) and sooty mangabey (SM).

due to negative regulation by IL10 (Chehimi *et al.*, 1994). The potential effects of HIV infection on the regulation of IL-12 production are complex and include regulation by IL-10, TGF-beta, IFN-gamma and other cytokines (reviewed by Ma and Montaner, 2000).

Analysis of IL-12p40 promoter sequences from RM and SM showed relatively high degree of variation among the cytokine promoters analyzed in this report (Table I). Thus the RM and SM derived promoter sequences exhibited 95.7% similarity to the human sequence. While the polymorphic sites were present throughout the sequence, the promoter region immediately upstream of the transcriptional start site (up to position −130) showed a relatively small degree of variability with only three identical single base substituions present in both NHP species (Fig. 2). On the contrary, the second region—spanning nt −130 up to position −510—showed multiple variations with several multinucleotide deletions being the most prominent. Thus there are two identical deleted regions at positions −136 and −168 (2 nt and 9 nt deletions, respectively) present in both NHP species. While neither of them targets a known TF binding site the latter one targets a region previously shown to be protected in DNAse footprinting assays suggesting the presence of yet an unknown TF (Becker *et al.*, 2001). There was also an A-T substitution in both NHP species at position −230 targeting an AP-1binding site. The promoter region further upstream, besides showing multiple single nucleotide substitutions present in both NHP species, also shows several sequence variation between the two NHP species. Notably, there is a single C-T substitutions at position −345 within the SM derived sequence that targets a binding site for the transcriptional factor Sp1. The third and the most distal promoter region analyzed (−510 to −730) shows several single bp substitutions and a deletions, where the C-G substitution at position −714 and a single nucleotide deletion at position −514 flanking the NFIL6 binding site are present only in the SM sequence. An additional single nucleotide variation that is present at position −642 is discordant between the two NHP species—e.g. the substitution is G-C in RM and G-T in SM. Taken together, the IL-12 p40 promoter sequence exhibited substantial variability between the species overall and particularly the two variations observed in the SM sequence that target binding sites

FIGURE 2 Sequence analysis of IL-12 p40 promoter. IL-12 p40 promoter sequences from SM and RM were aligned to the corresponding human sequence (HU). The numbering reflects positions in the human sequence. Highly variable region (− 130 to − 729) is shown; sequence deletions are marked by "x" and binding sites of selected TFs are underlined. "*" Marks a region protected in DNAse footprinting assays suggesting the presence of yet an unknown TF (Becker *et al.*, 2001).

for AP-1 and Sp1 can potentially play an important role in the transcriptional regulation of IL-12 and subsequently in the differences in the Th phenotype observed between the two NHP species.

### IFN-gamma Promoter

IFN-gamma production constitutes an essential part of Th1 mediated immune response in viral diseases. In lentivirus induced disease it was shown that IFN-gamma can restore cell mediated immunity in cells from HIV infected patients *in vitro* (De Francesco *et al.*, 1996). Interestingly however, Nicastri *et al.* documented not only a low Th1 cytokine production (including IFN-gamma) and increased IL-4 in HIV seropositive subjects but also low IFN-gamma and IL-2 in their HIV exposed seronegative partners suggesting that perhaps lower levels of these cytokines are associated with lower levels of cell activation and may confer a protective effect against infection (Nicastri *et al.*, 1999). Similarly in SIV model of AIDS in nonhuman primates, analysis of unstimulated expression of cytokines showed increase in IL-2 and IFN-gamma in RM but a similar increase of only IL-10 in SM after experimental infection with SIVmac239 in both species (Kaur *et al.*, 1998), providing evidence for an association between increased IFN-gamma levels with pathogenic SIV infection in RM. Yet another study by Orandle *et al.* showed a positive correlation between IFN-gamma levels and SIV infection with concurrent neuropathology (Orandle *et al.*, 2001).

Analysis of IFN-gamma regulatory sequences in a cohort of Caucasian HIV patients showed very little polymorphism, in fact the sequences upstream of TATA box (up to position − 777) were invariant in both HIV positive subjects and controls (Bream *et al.*, 2000). Interestingly, a recent study in an African–American cohort showed a positive association of − 179 G-T IFN-gamma promoter polymorphism with accelerated progression of HIV disease (An *et al.*, 2003).

Analysis of the proximal IFN-gamma promoter sequences (up to −750) showed >97% similarity between each of the NHP species and humans which represented the lowest degree of variation of all the promoter regions studied (Table I). The whole region contained only 11 identical single bp substitutions and two identical single bp insertions that were present in the two NHP species when compared to human sequence. The sequences from both NHP species also exhibited the highest degree of homology when compared to each other (99.6%) with each sequence containing only one single nt variation. Neither of the NHP sequences contained the −179 G-T polymorphism.

The low degree of variability between the IFN-gamma promoter sequences from the two species suggests it is unlikely that the observed differences in Th phenotype in these species are secondary to the sequence variations within the proximal promoter sequences.

### TNF-alpha Promoter

TNF-alpha is an important cytokine involved in HIV induced apoptosis of CD4+ T cells (Klein *et al.*, 1996) and was shown to play an important role in HIV gp120 mediated CD4+ T cell anergy (Kaneko *et al.*, 1997). Increased levels of serum TNF-alpha were shown to correlate with advanced HIV disease (Havlir *et al.*, 2001) and SIV infection in macaques (Benveniste *et al.*, 1996a). TNF-alpha was also shown to activate HIV-1 replication in infected cells *in vitro* (Folks *et al.*, 1989) and to function as a mediator of HIV tat induced activation of monocytes (Lafrenie *et al.*, 1997). Several polymorphisms of the TNF-alpha promoter sequence (G-A at positions −376, −308, −244 and −238) have been shown to associate with altered course of HCV infection (Lio *et al.*, 2003), malaria (May *et al.*, 2000) and leprosy (Santos *et al.*, 2002).

Potential effect of TNF-alpha promoter polymorphism in AIDS progression has been also studied and Quasney *et al.* showed a link between the presence of the A allele at position −308 and AIDS dementia (Quasney *et al.*, 2001). However, other reports failed to establish a link between this polymorphism and disease progression (Brinkman *et al.*, 1997; Knuchel *et al.*, 1998). Since we have previously showed that SM exhibit ~50% lower levels of serum TNF-alpha than humans or RM (Villinger *et al.*, 1993) it was of interest to investigate whether potential TNF-alpha promoter sequence polymorphisms could be associated with this phenotype. The analysis showed that the RM and SM TNF-alpha promoter sequences show the highest degree of diversity to (94.4% and 93.9% similarity the published human sequence, respectively) of all the promoter sequences analyzed. While the detected diversity of sequences is mostly caused by isolated single base substitutions or deletions that in most cases are present in both NHP species there are several regions in the promoter where the monkey sequences exhibit substantial degree of diversity between the two NHP species (Fig. 3).

Interestingly, both species show G-A polymorphisms at positions −376 and −308. While AP-1, AP-2 and Sp1 binding sites immediately upstream of TATA box are not targeted by any sequence variations, there is a 3 bp deletion in the region between the closely spaced Sp1 and AP-2 sites in both monkey species and an additional single bp substitution in SM sequence only (Fig. 3B). Sequence between positions −260 and −885 represents another variable region (Fig. 3A). The downstream part of this region (−467 to −630) shows five different insertion points and several substitutions. There are three 2 bp insertions at positions −467, −504 and −627 and one 1 bp insertion at position −538 (targeting the putative Sp1 binding site) that are identical in both monkey species.

However, there is also a discordant 2 bp insertion in RM sequence vs. 1 bp insertion in SM sequence at position −591 targeting putative c-Rel TF binding site. Another region with a high sequence diversity was detected in upstream promoter sequences (Fig. 3A, region −660 to −885). This region exhibited two deletions (positions −853 and −868) targeting the myeloid zinc-finger factor 1 TF (MZF1) binding site and flanking the NF-kappaB TF binding site that were identical in both monkeys, but SM sequence exhibited an extra single bp deletion at position −856. In addition, the SM sequence contained an additional single bp deletion (position −811), two 1 bp insertions (positions −738 and −767) and three 1 bp substitutions (−661, −704 and −741). Taken together, the sequences exhibit the lowest relative degree of homology overall and there are several regions exhibiting diverse sequence variations specific for SM that could be one of the mechanisms involved in the observed phenotype represented by the lower levels of TNF-alpha detected in the serum of this species.

### IL-10 Promoter

IL-10 is a potent cytokine involved in the regulation of Th type immune responses and increased IL-10 levels have been associated with rapid disease progression in HIV infected humans (Stylianou *et al.*, 1999) and with SIV infection in macaques (Benveniste *et al.*, 1996a). The increased levels of IL-10 were shown to negatively affect responses to recall antigens in HIV infected humans (Daftarian *et al.*, 1995), but on the other hand. IL-10 was also shown to inhibit HIV replication in human monocytes/macrophages (Akridge *et al.*, 1994). One of the mechanisms potential responsible for the differential IL-10 expression in HIV-1 infected individuals is the association of select polymorphisms within the IL-10 promoter with faster/delayed disease progression (Shin *et al.*, 2000). Analysis of IL-10 promoter sequences from RM revealed several sequence variations in upstream and downstream promoter sequences (Fig. 4A and B) identical to those previously described (Kumarvelu *et al.*, 2001) and that are present, in most cases in SM as well. Thus we observed a unique and extensive insertion of 9 nt sequence at position −715. Computer analysis

FIGURE 3 Sequence analysis of TNF-alpha promoter. TNF-alpha promoter sequences from SM and RM were aligned to the corresponding human sequence (HU). The numbering reflects positions in the human sequence. Highly variable regions upstream (A) and adjacent (B) to the TATA box are shown. Sequence deletions are marked by "x", insertions by " = " and binding sites of selected TFs are underlined. "*" Marks G-A polymorphisms at positions −308 and −376.

indicated that this inserted sequence could form a newly created binding site for N-myc. Interestingly, this sequence insertion is identical in both SM and RM monkeys—which may indicate that it probably is not one of the variations potentially underlying the observed differences in Th phenotype and/or SIV disease resistance/susceptibility. Also several point substitutions and/or insertions previously described were observed in both monkey species. We detected only one 3 bp deletion (position −255), rather than three deletions closely spaced together (Kumarvelu *et al.*, 2001), which is present in both NHP species. The Sp1 site (−631 to −637) previously described to be crucial for IL-10 induction following stimulation with LPS (Ma *et al.*, 2001) bears C-T polymorphism that is, however also present in both primate species. Neither monkey species exhibited the −592 polymorphism that was previously described to be associated with differential rate of AIDS disease progression in humans (Shin *et al.*, 2000).

Interestingly we detected several polymorphic sites within the IL-10 promoter that were represented by single nucleotide substitutions that were present in SM derived sequences, but not in RM or human derived promoters. Specifically there was G-A substitution in SM at position −864 that targets the center of one of the C/EBP binding sites previously shown to be important for cAMP mediated upregulation of IL-10 transcription (Brenner *et al.*, 2003). More importantly, two observed single base substitutions (T-C at position −804 and C-T at position −562) created two potential binding sites for the transcriptional factor p300. In addition a G-A substitution at position −949 created a putative binding site for Oct1 and A-C substitution at position −750 created a putative binding site for MZF1.

FIGURE 4 Sequence analysis of IL-10 promoter. IL-10 promoter sequences from SM and RM were aligned to the corresponding human sequence (HU). The numbering reflects positions in the human sequence. Highly variable upstream (A) and downstream (B) promoter regions are shown. Sequence deletions are marked by "x", insertions by " = " and binding sites of selected TFs are underlined. Underlined bases in SM derived sequence indicate predicted biding site created by sequence variation. "*" Marks G-A polymorphisms at positions −308 and −376.

Taken together, while most of the variations observed in RM compared with the human derived sequence are also present in the SM monkeys, there are several discrete polymorphisms specific for SM derived sequence that—although primarily consisting only of single nucleotide substitutions—create putative binding sites for well described TFs that may contribute to the differential regulation of IL-10 expression with the observed skew in the Th type response.

### IL-4 Promoter

IL-4 is one of the major cytokines involved in Th2 polarization (Parronchi *et al.*, 1992) and has been shown to downregulate IL-12p40 (Th1 cytokine) production at both the transcriptional and the post-transcriptional levels (Seegmuller *et al.*, 2003). Several studies have addressed the potential role of IL-4 promoter polymorphisms and IL-4 levels in HIV disease. Thus decrease of IL-4 (and IFN-gamma) levels was found to be associated with neurological complications in AIDS patients (Wesselingh *et al.*, 1994). While one study found the −590 C-T polymorphism was associated with HIV disease (Smolnikova and Konenkov, 2002), the results from another study suggested that this polymorphism was associated with a delayed acquisition of CXCR4 tropic HIV variants, but with no effect on disease progression (Kwa *et al.*, 2003).

In our analysis of the protype Th1/Th2 cytokines in RM and SM we found that PHA stimulated PBMC from SM express significantly higher levels of IL-4 when compared with similar cells from humans or RM (Fig. 1). However, when we cloned and sequenced the IL-4 promoter from each of the two nonhuman primate species, we found a relatively high degree of homology between the sequences from different species (Table I). Specifically, very little sequence variation was observed within the very proximal promoter sequences—e.g. the sequences were completely homologous up to position −200 upstream of the start site and exhibited ~98.2% homology up to position −540. Within this region all the variations found are present in both RM and SM (compared to human sequence) and none

FIGURE 5 Sequence analysis of IL-4 promoter. IL-4 promoter sequences from SM and RM were aligned to the corresponding human sequence (HU). The numbering reflects positions in the human sequence. Highly variable upstream (A) and downstream (B) promoter regions are shown. Sequence deletions are marked by "x", insertions by " = " and binding sites of selected TFs are underlined. Underlined bases in SM derived sequence indicate predicted biding site created by sequence variation.

of the variations targets a described TF binding site (Fig. 5B). On the contrary, sequences between positions −540 and −950 exhibit substantial variations (~10%) between the nonhuman primate and human sequences and some of this variation is discordant between the SM and RM (Fig. 5A). Notably there is an insertion at position −554 in the SM sequence that creates a potential binding site for C/EBP, a TF previously shown to play an important role in the regulation of IL-4 transcription (Berberich-Siebelt *et al.*, 2000). Moreover, there is a significant insertion in both RM and SM (3 and 4 bp, respectively) within the poly A region at position −670. The region further upstream in the NHP sequences contains numerous single bp substitutions (where two are discordant between SM vs. RM and/or humans) as well as four 1 single bp insertion and one 2 bp deletion. The −590 site does not show any variation in these two NHP species. Taken together, the fact that the proximal IL4 promoter from each of the species exhibited a relatively high degree of homology seems to indicate that the observed species specific differences in IL-4 expression are probably not based on a genetic predisposition leading to the differential binding of the appropriate TF. However, it is possible that the creation of the additional C/EBP binding site can positively affect IL-4 transcription in SM.

### Acknowledgements

This work was supported by NIH RO1 AI51994.

### References

Ahmed, R.K., Makitalo, B., Karlen, K., Nilsson, C., Biberfeld, G. and Thorstensson, R. (2002) "Spontaneous production of RANTES and antigen-specific IFN-gamma production in macaques vaccinated with SHIV-4 correlates with protection against SIVsm challenge", *Clin. Exp. Immunol.* **129**, 11–18.

Akridge, R.E., Oyafuso, L.K. and Reed, S.G. (1994) "IL-10 is induced during HIV-1 infection and is capable of decreasing viral replication in human macrophages", *J. Immunol.* **153**, 5782–5789.

An, P., Vlahov, D., Margolick, J.B., *et al.* (2003) "A tumor necrosis factor-alpha-inducible promoter variant of interferon-gamma accelerates CD4+T cell depletion in human immunodeficiency virus-1-infected individuals", *J. Infect. Dis.* **188**, 228–231.

Ansari, A.A., Mayne, A., Hunt, D., Sundstrom, J.B. and Villinger, F. (1994) "TH1/TH2 subset analysis. I. Establishment of criteria for subset identification in PBMC samples from nonhuman primates", *J. Med. Primatol.* **23**, 102–107.

Becker, C., Wirtz, S., Ma, X., Blessing, M., Galle, P.R. and Neurath, M.F. (2001) "Regulation of IL-12 p40 promoter activity in primary human monocytes: roles of NF-kappaB, CCAAT/enhancer-binding protein beta, and PU.1 and identification of a novel repressor element (GA-12) that responds to IL-4 and prostaglandin E(2)", *J. Immunol.* **167**, 2608–2618.

Benveniste, O., Vaslin, B., Le Grand, R., *et al.* (1996a) "Interleukin 1 beta, interleukin 6, tumor necrosis factor alpha, and interleukin 10 responses in peripheral blood mononuclear cells of cynomolgus macaques during acute infection with SIVmac251", *AIDS Res. Hum. Retrovir.* **12**, 241–250.

Benveniste, O., Vaslin, B., Le Grand, R., *et al.* (1996b) "Comparative interleukin (IL-2)/interferon IFN-gamma and IL-4/IL-10 responses during acute infection of macaques inoculated with attenuated nef-truncated or pathogenic SICmac251 virus", *Proc. Natl Acad. Sci. USA* **93**, 3658–3663.

Berberich-Siebelt, F., Klein-Hessling, S., Hepping, N., *et al.* (2000) "C/EBPbeta enhances IL-4 but impairs IL-2 and IFN-gamma induction in T cells", *Eur. J. Immunol.* **30**, 2576–2585.

Bream, J.H., Carrington, M., O'Toole, S., *et al.* (2000) "Polymorphisms of the human IFNG gene noncoding regions", *Immunogenetics* **51**, 50–58.

Brenner, S., Prosch, S., Schenke-Layland, K., Riese, U., Gausmann, U. and Platzer, C. (2003) "cAMP-induced Interleukin-10 promoter activation depends on CCAAT/enhancer-binding protein expression and monocytic differentiation", *J. Biol. Chem.* **278**, 5597–5604.

Brinkman, B.M., Keet, I.P., Miedema, F., Verweij, C.L. and Klein, M.R. (1997) "Polymorphisms within the human tumor necrosis factor-alpha promoter region in human immunodeficiency virus type 1-seropositive persons", *J. Infect. Dis.* **175**, 188–190.

Chehimi, J., Starr, S.E., Frank, I., *et al.* (1994) "Impaired interleukin 12 production in human immunodeficiency virus-infected patients", *J. Exp. Med.* **179**, 1361–1366.

Clerici, M., Balotta, C., Meroni, L., *et al.* (1996) "Type 1 cytokine production and low prevalence of viral isolation correlate with long-term nonprogression in HIV infection", *AIDS Res. Hum. Retrovir.* **12**, 1053–1061.

Daftarian, M.P., Diaz-Mitoma, F., Creery, W.D., Cameron, W. and Kumar, A. (1995) "Dysregulated production of interleukin-10 (IL-10) and IL-12 by peripheral blood lymphocytes from human immunodeficiency virus-infected individuals is associated with altered proliferative responses to recall antigens", *Clin. Diagn. Lab. Immunol.* **2**, 712–718.

De Francesco, M.A., Caruso, A., Dima, F., *et al.* (1996) "IFN-gamma restores HIV- and non-HIV-specific cell mediated immune response *in vitro* and its activity is neutralized by antibodies from patients with AIDS", *Scand. J. Immunol.* **43**, 94–100.

Folks, T.M., Clouse, K.A., Justement, J., *et al.* (1989) "Tumor necrosis factor alpha induces expression of human immunodeficiency virus in a chronically infected T-cell clone", *Proc. Natl Acad. Sci. USA* **86**, 2365–2368.

Fultz, P.N., McClure, H.M., Anderson, D.C., Swenson, R.B., Anand, R. and Srinivasan, A. (1986) "Isolation of a T-lymphotropic retrovirus from naturally infected sooty mangabey monkeys (Cercocebus atys)", *Proc. Natl Acad. Sci. USA* **83**, 5286–5290.

Gubler, U., Chua, A.O., Schoenhaut, D.S., *et al.* (1991) "Coexpression of two distinct genes is required to generate secreted bioactive cytotoxic lymphocyte maturation factor", *Proc. Natl Acad. Sci. USA* **88**, 4143–4147.

Havlir, D.V., Torriani, F.J., Schrier, R.D., *et al.* (2001) "Serum interleukin-6 (IL-6), IL-10, tumor necrosis factor (TNF) alpha, soluble type II TNF receptor, and transforming growth factor beta levels in human immunodeficiency virus type 1-infected individuals with Mycobacterium avium complex disease", *J. Clin. Microbiol.* **39**, 298–303.

Heinemeyer, T., Wingender, E., Reuter, I., *et al.* (1998) "Databases on transcriptional regulation: TRANSFAC, TRRD and COMPEL", *Nucleic Acids Res.* **26**, 362–367.

Kaneko, H., Hishikawa, T., Sekigawa, I., Hashimoto, H., Okumura, K. and Kaneko, Y. (1997) "Role of tumour necrosis factor-alpha (TNF-alpha) in the induction of HIV-1 gp120-mediated CD4+ T cell anergy", *Clin. Exp. Immunol.* **109**, 41–46.

Kaur, A., Grant, R.M., Means, R.E., McClure, H., Feinberg, M. and Johnson, R.P. (1998) "Diverse host responses and outcomes following simian immunodeficiency virus SIVmac239 infection in sooty mangabeys and rhesus macaques", *J. Virol.* **72**, 9597–9611.

Klein, S.A., Dobmeyer, J.M., Dobmeyer, T.S., *et al.* (1996) "TNF-alpha mediated apoptosis of CD4 positive T-lymphocytes. A model of T-cell depletion in HIV infected individuals", *Eur. J. Med. Res.* **1**, 249–258.

Klein, S.A., Dobmeyer, J.M., Dobmeyer, T.S., *et al.* (1997) "Demonstration of the Th1 to Th2 cytokine shift during the course of HIV-1 infection using cytoplasmic cytokine detection on single cell level by flow cytometry", *AIDS* **11**, 1111–1118.

Knuchel, M.C., Spira, T.J., Neumann, A.U., *et al.* (1998) "Analysis of a biallelic polymorphism in the tumor necrosis factor alpha promoter and HIV type 1 disease progression", *AIDS Res. Hum. Retrovir.* **14**, 305–309.

Kumarvelu, J., Shanmugasundaram, G.K., Unwalla, H., Ramamoorti, N. and Banerjea, A.C. (2001) "Genetic analyses of the promoter region of interleukin-10 gene in different species of monkeys: implications for HIV/AIDS progression", *Genes Immun.* **2**, 404–407.

Kwa, D., van Rij, R.P., Boeser-Nunnink, B., Vingerhoed, J. and Schuitemaker, H. (2003) "Association between an interleukin-4 promoter polymorphism and the acquisition of CXCR4 using HIV-1 variants", *AIDS* **17**, 981–985.

La Frenie, R.M., Wahl, L.M., Epstein, J.S., Yamada, K.M. and Dhawan, S. (1997) "Activation of monocytes by HIV-Tat treatment is mediated by cytokine expression", *J. Immunol.* **159**, 4077–4083.

Lio, D., Caruso, C., Di Stefano, R., *et al.* (2003) "IL-10 and TNF-alpha polymorphisms and the recovery from HCV infection", *Hum. Immunol.* **64**, 674–680.

Ma, X. and Montaner, L.J. (2000) "Proinflammatory response and IL-12 expression in HIV-1 infection", *J. Leukoc. Biol.* **68**, 383–390.

Ma, W., Lim, W., Gee, K., *et al.* (2001) "The p38 mitogen-activated kinase pathway regulates the human interleukin-10 promoter via the activation of Sp1 transcription factor in lipopolysaccharide-stimulated human macrophages", *J. Biol. Chem.* **276**, 13664–13674.

Maggi, E., Mazzetti, M., Ravina, A., *et al.* (1994) "Ability of HIV to promote a TH1 to TH0 shift and to replicate preferentially in TH2 and TH0 cells", *Science* **265**, 244–248.

Manetti, R., Parronchi, P., Giudizi, M.G., *et al.* (1993) "Natural killer cell stimulatory factor (interleukin 12 [IL-12]) induces T helper type 1 (Th1)-specific immune responses and inhibits the development of IL-4-producing Th cells", *J. Exp. Med.* **177**, 1199–1204.

May, J., Lell, B., Luty, A.J., Meyer, C.G. and Kremsner, P.G. (2000) "Plasma interleukin-10: Tumor necrosis factor (TNF)-alpha ratio is associated with TNF promoter variants and predicts malarial complications", *J. Infect. Dis.* **182**, 1570–1573.

Mingari, M.C., Maggi, E., Cambiaggi, A., *et al.* (1996) "Development *in vitro* of human CD4+ thymocytes into functionally mature Th2 cells. Exogenous interleukin-12 is required for priming thymocytes to produce both Th1 cytokines and interleukin-10", *Eur. J. Immunol.* **26**, 1083–1087.

Nakayama, E.E., Hoshino, Y., Xin, X., *et al.* (2000) "Polymorphism in the interleukin-4 promoter affects acquisition of human immunodeficiency virus type 1 syncytium-inducing phenotype", *J. Virol.* **74**, 5452–5459.

Nicastri, E., Sarmati, L., Ercoli, L., *et al.* (1999) "Reduction of IFN-gamma and IL-2 production by peripheral lymphocytes of HIV-exposed seronegative subjects", *AIDS* **13**, 1333–1336.

Orandle, M.S., Williams, K.C., MacLean, A.G., Westmoreland, S.V. and Lackner, A.A. (2001) "Macaques with rapid disease progression and simian immunodeficiency virus encephalitis have a unique cytokine profile in peripheral lymphoid tissues", *J. Virol.* **75**, 4448–4452.

Parronchi, P., De Carli, M., Manetti, R., *et al.* (1992) "IL-4 and IFN (alpha and gamma) exert opposite regulatory effects on the development of cytolytic potential by Th1 or Th2 human T cell clones", *J. Immunol.* **149**, 2977–2983.

Quasney, M.W., Zhang, Q., Sargent, S., Mynatt, M., Glass, J. and McArthur, J. (2001) "Increased frequency of the tumor necrosis factor-alpha-308 A allele in adults with human immunodeficiency virus dementia", *Ann. Neurol.* **50**, 157–162.

Santos, A.R., Suffys, P.N., Vanderborght, P.R., *et al.* (2002) "Role of tumor necrosis factor-alpha and interleukin-10 promoter gene polymorphisms in leprosy", *J. Infect. Dis.* **186**, 1687–1691.

Seegmuller, I., Hacker, H. and Wagner, H. (2003) "IL-4 regulates IL-12 p40 expression post-transcriptionally as well as via a promoter-based mechanism", *Eur. J. Immunol.* **33**, 428–433.

Shin, H.D., Winkler, C., Stephens, J.C., *et al.* (2000) "Genetic restriction of HIV-1 pathogenesis to AIDS by promoter alleles of IL10", *Proc. Natl Acad. Sci. USA* **97**, 14467–14472.

Smolnikova, M.V. and Konenkov, V.I. (2002) "Association of IL2, TNFA, IL4 and IL10 Promoter Gene Polymorphisms with the Rate of Progression of the HIV Infection", *Russ. J. Immunol.* **7**, 349–356.

Stylianou, E., Aukrust, P., Kvale, D., Muller, F. and Froland, S.S. (1999) "IL-10 in HIV infection: increasing serum IL-10 levels with disease progression–down-regulatory effect of potent anti-retroviral therapy", *Clin. Exp. Immunol.* **116**, 115–120.

Villinger, F., Hunt, D., Mayne, A., Vuchetich, M., Findley, H. and Ansari, A.A. (1993) "Qualitative and quantitative studies of cytokines synthesized and secreted by non-human primate peripheral blood mononuclear cells", *Cytokine* **5**, 469–479.

Wesselingh, S.L., Glass, J., McArthur, J.C., Griffin, J.W. and Griffin, D.E. (1994) "Cytokine dysregulation in HIV-associated neurological disease", *Adv. Neuroimmunol.* **4**, 199–206.

